# Plasma sphingomyelins are associated with plasma total‐Tau in presymptomatic Alzheimer's disease

**DOI:** 10.1002/alz70856_101826

**Published:** 2025-12-24

**Authors:** Tahmida Sharmin, Pratishtha Chatterjee, James D. Doecke, Nicholas Ashton, Hamid R Sohrabi, Henrik Zetterberg, Kaj Blennow, Manohar Garg, Ralph N Martins

**Affiliations:** ^1^ Macquarie Medical School, Macquarie University, Macquarie Park, NSW, Australia; ^2^ Department of Pharmacy, University of Rajshahi, Rajshahi, Bangladesh; ^3^ Florey Institute of Neuroscience and Mental Health, University of Melbourne, Melbourne, VIC, Australia; ^4^ School of Medical and Health Sciences, Edith Cowan University, Perth, Western Australia, Australia; ^5^ The Australian e‐Health Research Centre, CSIRO, Brisbane, QLD, Australia; ^6^ Department of Old Age Psychiatry, Institute of Psychiatry, Psychology & Neuroscience, King's College London, London, United Kingdom; ^7^ Department of Psychiatry and Neurochemistry, Institute of Neuroscience and Physiology, The Sahlgrenska Academy, University of Gothenburg, Mölndal, Sweden; ^8^ School of Psychology, Murdoch University, Murdoch, Western Australia, Australia; ^9^ Alzheimer's Research Australia, Perth, Western Australia, Australia; ^10^ Clinical Neurochemistry Laboratory, Sahlgrenska University Hospital, Mölndal, Västra Götalands län, Sweden; ^11^ Department of Neurodegenerative Disease, UCL Institute of Neurology, Queen Square, London, United Kingdom; ^12^ Wisconsin Alzheimer's Disease Research Center, University of Wisconsin‐Madison, School of Medicine and Public Health, Madison, WI, USA; ^13^ Department of Psychiatry and Neurochemistry, University of Gothenburg, Gothenburg, Sweden; ^14^ UK Dementia Research Institute, University College London, London, United Kingdom; ^15^ Hong Kong Center for Neurodegenerative Diseases, Hong Kong, Hong Kong, China; ^16^ Clinical Neurochemistry Laboratory, Sahlgrenska University Hospital, Mölndal, Sweden; ^17^ Macquarie University, Sydney, NSW, Australia

## Abstract

**Background:**

Altered plasma sphingomyelin levels have been associated with Alzheimer's disease (AD), indicating disruptions in lipid metabolism and their potential link with AD pathogenesis. Meanwhile, tau‐related neuronal damage and neurodegeneration result in the release of neuronal tau protein into the bloodstream, reflected predominantly as plasma total‐Tau. Exploring the relationship between sphingomyelin metabolism and tau release before symptom onset could offer valuable insights into the biochemical pathways linked to AD and support the development of improved diagnostic and therapeutic strategies.

**Method:**

The current study embraced plasma sample analysis using a Biocrates‐based targeted mass‐spectrometry platform to measure sphingomyelin levels and a single‐molecule array (Simoa) platform to quantify total‐Tau levels. The participants involved in this study were from the KARVIAH cohort —100 cognitively normal (CN; MoCA≥26 & MMSE≥26) older adults who underwent positron emission tomography (PET) to assess cortical amyloid‐beta (A*β*) load and were grouped as CN A*β*‐ (normal cortical‐A*β* load) and CN A*β*+ (higher cortical‐A*β* load). This study examined cross‐sectional correlations between plasma sphingomyelins and total‐Tau levels within each group. Sphingomyelins associated with total‐Tau were further analysed for their relationship with cortical‐Aβ load. All statistically significant correlations were assessed for correction for AD‐related confounding variables, containing gender, age, body mass index (BMI), and *APOE* ε4 status, with additional amendments for the false discovery rate (FDR).

**Result:**

Statistically significant positive correlations between sphingomyelins and total‐Tau levels were observed exclusively in CN A*β*+ individuals. These associations remained robust after adjusting for AD‐related confounding variables and correcting for the FDR. Further analysis revealed significant inverse associations between total‐Tau‐associated sphingomyelins and cortical‐A*β* load, observed only in CN A*β*+ individuals, both with and without adjustment for confounding variables and FDR correction.

**Conclusion:**

Elevated plasma total‐Tau levels were associated with higher plasma sphingomyelins exclusively in CN A*β*+ individuals, suggesting a link between tau release from neuronal damage and sphingomyelin‐associated biochemical pathways in the presymptomatic stage of AD. Furthermore, higher levels of total‐Tau‐associated sphingomyelins in plasma were correlated with lower cortical‐A*β* load in CN A*β*+ individuals, likely reflecting early sphingomyelin‐mediated compensatory mechanisms against AD pathogenesis. These findings highlight the potential of total‐Tau‐associated sphingomyelins as markers for understanding the mechanisms involved in presymptomatic AD.

